# Treatment Resistant Early-Onset Schizophrenia: A Tale of Two Siblings

**DOI:** 10.1192/j.eurpsy.2024.930

**Published:** 2024-08-27

**Authors:** E. Yerlikaya Oral

**Affiliations:** Department of Child and Adolescent Psychiatry, University of Health Sciences, Bakirkoy Prof. Dr. Mazhar Osman Research and Training Hospital for Psychiatry, Neurology and Neurosurgery, Istanbul, Türkiye

## Abstract

**Introduction:**

Early-Onset Schizophrenia (EOS) is a rare and severe form of schizophrenia that begins in childhood and it is often associated with genetic risk factors, poorer prognosis, and increased treatment resistance compared to adult-onset schizophrenia (Hatzimanolis *et al.* Eur Psychiatry 2020;63(1):e44). This case report presents two siblings diagnosed with EOS and treated at the same inpatient clinic in different years.

**Objectives:**

The aim is offering a perspective on the clinical characteristics, genetic and enviromental implications, and treatment challenges of two siblings with EOS and seeking to enhance understanding of EOS’s complexity, particularly in the context of treatment resistance.

**Methods:**

A comprehensive retrospective review of the siblings’ all medical records was conducted, focusing on their psychiatric history, symptoms, treatment trials, and responses of treatment. Both cases’ current clinical situations were evaluated cross-sectionally.

**Results:**

*Older sibling:* 19 year-old male, was diagnosed with EOS following the onset of symptoms as social withdrawal, negativism and suspiciousness at the age of 14. He referred to the inpatient clinic with the cause of drug intake refusal. Risperidone treatment started but there was no significant response. Risperidone to olanzapine switch made and clinical remission observed. After his discharge, 4 more hospitalisations in 5 years needed due to low socioeconomic status, parental neglect and him having no insight and stopped taking his medications repeatedly. Several depot form antipsychotic injections started to prevent recurrent hospitalisation. Despite that he needed several hospitalisations to adult psychiatry inpatient clinics.*Younger sibling:* 14 year-old female, were diagnosed with EOS following the symptoms as auditory hallucinations, suspiciousness, disorganised speech and behaviours at the age of 13. She referred to the same inpatient clinic with suicidal risk after 2 years of his brother’s last hospital stay. She responded good to olanzapine treatment like her brother’s, during her first stay. After 2 weeks of her discharge, her psychotic symptoms started again with no specific reason. Second hospitalisation needed due to her homicidal and suicidal risk. Clozapine and aripipirazole treatment started and she discharged in partial remission. She is being followed in outpatient clinic, with low functioning.

**Conclusions:**

Despite trials of multiple antipsychotic medications and adjunctive treatments, both siblings demonstrated significant treatment resistance. These sibling cases underscore the complexity and challenges in managing EOS, particularly when it presents with treatment resistance. The shared familial environment and potential genetic factors demand further investigation to elucidate the pathogenesis of EOS and optimize therapeutic approaches.

**Disclosure of Interest:**

None Declared

